# Advances in programmable DNA nanostructures enabling stimuli-responsive drug delivery and multimodal biosensing

**DOI:** 10.1039/d5cb00057b

**Published:** 2025-06-17

**Authors:** Yao Hong, Wenyue Ma, Meixia Wang, Hong-Hui Wang

**Affiliations:** a State Key Laboratory of Chemo and Biosensing, College of Biology, Hunan Provincial Key Laboratory of Biomacromolecular Chemical Biology, Hunan University Changsha 410082 P. R. China wangmeixia@hnu.edu.cn wanghonghui@hnu.edu.cn

## Abstract

Recent advancements in DNA nanotechnology have unlocked unprecedented opportunities to address critical challenges in precision medicine, particularly in targeted drug delivery and biomedical imaging. Conventional nanocarriers often suffer from poor spatiotemporal control, suboptimal tumor accumulation, and non-specific biodistribution. To overcome these limitations, DNA-engineered nanostructures—including tile-based assemblies, origami frameworks, spherical nucleic acids, and stimuli-responsive hydrogels—have emerged as programmable platforms capable of dynamically responding to tumor microenvironmental cues (*e.g.*, pH, enzymatic activity, redox gradients) for triggered drug release. In this review, we comprehensively analyze these architectures with emphasis on their modular design strategies, *in vivo* stability improvements *via* polyethylene glycol (PEG) functionalization, and multi-ligand targeting capabilities against cancer-specific biomarkers. In addition to therapeutic uses, these nanostructures also enable highly sensitive detection of circulating tumor DNA and exosomes using fluorescence resonance energy transfer (FRET) probes, electrochemiluminescence amplification circuits, SERS substrates, and cell variable region sensing technology. They also allow for real-time monitoring of dynamic intercellular interactions, overcoming the constraints of traditional sensing methods. This review systematically elaborates on the structural characteristics of DNA assemblies and summarizes the innovative applications of these nanostructures in multimodal detection, offering a more comprehensive perspective for early cancer diagnosis and precision treatment. Despite promising preclinical results, key translational challenges persist, including scalable manufacturing bottlenecks, immune compatibility optimization, and rigorous assessment of long-term nanotoxicity. Future integration with artificial intelligence-driven design tools may catalyze the development of next-generation theranostic nanodevices, ultimately bridging the gap between synthetic biology and clinical oncology.

## Abstract 2

Programmable DNA nanostructures enable stimuli-responsive drug delivery and multimodal biosensing. This review highlights their modular design, stability, and precision in disease-targeted therapy and early cancer diagnosis.

## Graphical abstract

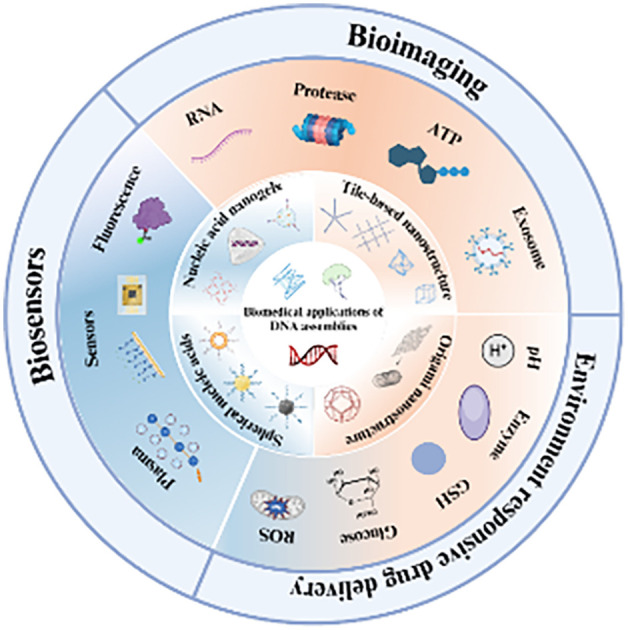

## 1. Introduction

In recent years, DNA nanomedicine has emerged in the field of tumor therapy, and DNA nanomaterials have been widely used in biomedicine because of their unique physicochemical properties due to their characteristic nanoscale dimensions (1–100 nm).^1^ Nanoscale DNA structures and functional materials are designed and synthesized using the properties of DNA, *i.e.*, based on DNA's precise base-pairing rules, which enable the formation of stable and functionally specific nanostructures. These materials have great potential in the field of nanotechnology due to their biocompatibility, programmability, low toxicity, and degradability.^2^

Drug molecules enter the body mainly by simple diffusion, which is often kinetically limited, and are easily degraded and cleared in the blood circulation, making it difficult to internalize them into cells or break them down in the cellular microenvironment.^3^ Electrostatic repulsion between anionic drug molecules and the negatively charged phospholipid bilayer makes it difficult for them to cross the cell membrane and limit their access to the cell.^4^ Therefore, the precise delivery of drug molecules to ideal sites such as tumor cells is challenging. Adjusting the pharmacological properties of the drug (such as half-life, biodistribution, and target concentration) to minimize enzymatic degradation and off-target effects is crucial. Pharmacological strategies can improve the bioavailability of drugs, prolong the biological half-life of drugs, and alter the absorption and release characteristics of drugs by altering the drug's form by preparing it into liposomes,^5^ micelles,^6^ and polymers.^7^ However, traditional drug delivery systems exhibit several limitations. For instance, liposomes were once regarded as a highly promising drug delivery platform due to their excellent biocompatibility and high drug-loading capacity.^8^ However, they tend to undergo fluidization and leakage at physiological temperatures, resulting in premature drug release.^9^ Moreover, stabilization strategies for liposomes based on charge interactions and chemical reagents may increase their toxicity and side effects. For example, cationic liposomes tend to accumulate in the spleen and liver, exerting toxicity on macrophages and organs.^10^ Polyethylene glycol (PEG)-modified liposomes may accelerate blood clearance and induce allergic reactions. Additionally, liposomes face various manufacturing challenges, including poor batch-to-batch reproducibility, insufficient drug encapsulation efficiency, limited scalability of sterilization methods, and high production costs, all of which restrict their widespread application.^11^ Polymer micelles and polymeric nanoparticles are two other common drug delivery systems. Polymer micelles are composed of amphiphilic block copolymers containing hydrophilic and hydrophobic monomer units, which can solubilize poorly water-soluble drugs, accumulate in regions with leaky vasculature, enhance drug bioavailability, and improve targeting capability through the conjugation of targeting ligands.^12^ However, their preparation process is complex, and the available materials are relatively limited, making it difficult to ensure consistency and large-scale production.^13^ Polymeric nanoparticles are synthesized from biodegradable or non-biodegradable polymers, and their drug-loading capacity and release profiles can be modulated by adjusting the polymer's molecular weight and crystallinity. Nevertheless, the polydispersity of polymeric nanoparticles significantly increases the likelihood of inconsistent manufacturing, and their potential toxicity remains a concern.^14^

To address these challenges, researchers have been exploring endogenous biomaterials. DNA, as an endogenous substance, possesses advantages such as bioactivity, non-toxicity, and biodegradability. Its unique molecular assembly patterns endow DNA nanostructures with precise programmability and excellent addressability, which has facilitated the development of more sophisticated DNA architectures and demonstrated their tremendous potential in the field of tumor nanomedicine.^15^ Several groups have reported studies using DNA nanostructures as drug carriers. However, unassembled DNA molecules have difficulty entering cells due to their negative charge and electrostatic repulsion with the cell membrane, but DNA assemblies are able to smoothly cross the cell membrane and enter cells without relying on transfection reagents, which provides new possibilities for the construction of safe and non-viral DNA nanocarriers for precise drug delivery. The specific mechanism by which the DNA assembly enters the cell is not completely clear. Fan *et al.*^16^ found that DNA assemblies can enter the cell rapidly through endocytosis mediated by the foveolar proteins and escape from the lysosomes into the nucleus after modification, and this process is similar to viral invasion of a cell. In addition, the affinity between DNA assemblies and scavenger receptors (SRs) on the cell membrane is critical for cellular uptake, and DNA assemblies of different shapes (*e.g.*, tetrahedral, trigonal, and cubic) differ in their ability to bind to scavenger receptors, affecting the frequency of cellular internalization.^17^ Similarly, spherical nucleic acids (SNAs) can achieve rapid cellular uptake and internal translocation by targeting class A scavenger receptors through lipid raft- and niche-dependent pathways, without the requirement of additional transfection means, due to their high affinity for complementary sequences and unique 3D structures.^18^ Researchers have capitalized on these advantages to develop a variety of DNA assemblies to create smart drug delivery systems that efficiently load anticancer drugs and deliver them precisely to the target area, significantly improving therapeutic efficacy.

The tumor microenvironment (TME) constitutes a complex cellular and molecular network that is of critical significance for tumor development. The TME characteristically exhibits hypoxia, an acidic environment, abnormal vascular proliferation, and numerous immunosuppressive cells, which limit the efficacy of tumor therapy.^19,20^ DNA assemblies, as drug delivery carriers, can be modified with multi-functional nanodevices at their spatial sites to achieve precise drug release in response to specific stimuli (*e.g.*, pH change, enzyme activity, endogenous molecules, *etc.*). With their stability and dynamic response properties, DNA assemblies show great potential in the biomedical field, especially in biomolecular sensing and imaging. They can trigger conformational changes through external stimuli, and then enhance signaling and logic operations through optical and electrochemical techniques. In addition, functionalized DNA assemblies (*e.g.*, with the addition of fluorophores or metal particles, *etc.*) can also be involved in drug tracking and assessment of therapeutic effects through visual imaging of endogenous substances, which provides a new strategy for precision therapy.

**Fig. 1:**
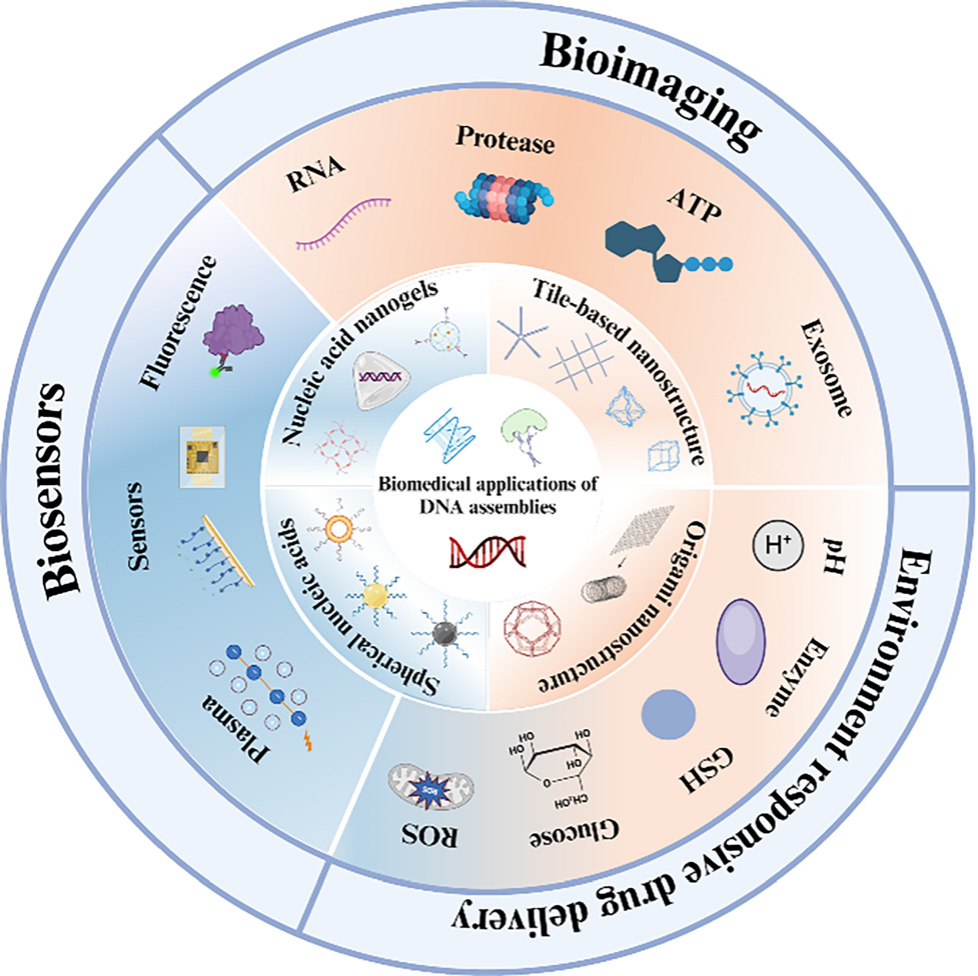
Four DNA assemblage structures, including DNA tile porcelain, DNA origami, spherical nucleic acids, and DNA hydrogels, for biomedical applications (figure produced in BioRender).

In the first part of this paper, the structural evolution of four representative DNA assemblies (*i.e.*, DNA tiles, DNA origami, spherical nucleic acids, and DNA hydrogels) is reviewed and summarized; in the second part, new strategies for drug delivery platforms as dynamically responsive carriers are highlighted; and in the third part, the latest applications of DNA assemblies in biomedical fields are analyzed and commented on, which are mainly related to their applications in sensing (*e.g.*, fluorescence, electrochemical sensing) and imaging of endogenous substance (Fig. 1). Finally, the current challenges of DNA assemblies and the corresponding solutions are discussed in detail, and the future development direction and application prospects are envisioned.

## 2. Structural advances in DNA assemblies

### 2.1. Tile-based DNA nanostructures

**Fig. 2:**
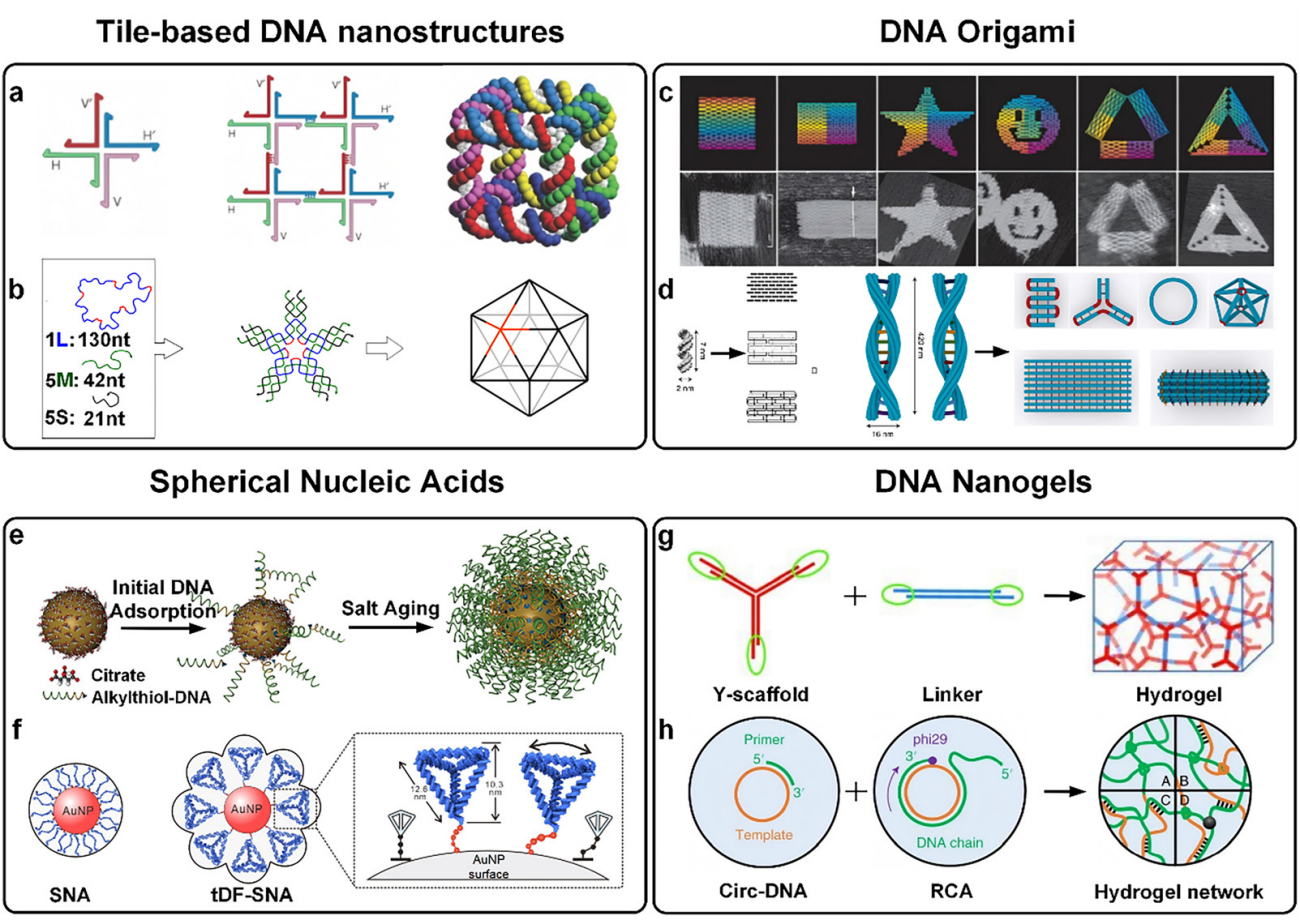
Structural advances in four DNA assemblies. (a) Two juxtaposed strands of double-helical DNA cross to form a cross-structured four-armed junction and are assembled by repetition into 2D and 3D structures. Reproduced with permission from ref. 22. Copyright 2003, Springer Nature Limited. (b) Three strands of DNA single strands are assembled into five-point star-shaped tiles with symmetrically banded sticky ends, which are further assembled into icosahedra. Reproduced with permission from ref. 30. Copyright 2008, National Academy of Sciences, U.S.A. (c) 2D symmetric patterns formed by DNA origami. Reproduced with permission from ref. 32. Copyright 2006, Springer Nature Limited. (d) Different DNA strands form M-DNA by complementary base pairing between the strands. Reproduced with permission from ref. 38. Copyright 2020, The Author(s), under exclusive license to Springer Nature Limited. (e) SNAs were formed by incubating AuNPs as the core with oligonucleotides that contained sulfhydryl groups (–SH) in a citrate environment. Reproduced with permission from ref. 45. Copyright 2012, American Chemical Society. (f) Self-assembled tDF-SNA was formed by inserting tetrahedral tDF into the SNA surface. Reproduced with permission from ref. 49. Copyright 2024, WILEY-VCH GmbH. (g) DNA hydrogels were formed by self-assembly using Y scaffolds hybridized to the sticky ends of the junctions. Reproduced with permission from ref. 53. Copyright 2011, WILEY-VCH Verlag GmbH & Co. KGaA, Weinheim. (h) DNA rolling ring replication synthesizes ultra-long DNA strands, which are physically cross-linked to form DNA hydrogels. Reproduced with permission from ref. 54. Copyright 2021, The Author(s), under exclusive license to Springer Nature Limited.

In DNA nanotechnology, the tile-based DNA nanostructure is a basic unit in which tiles are connected by complementary pairing of DNA sequences to obtain structural bodies on a larger scale. In 1982, inspired by Hooliday's model, Seeman used four DNA single strands to construct a cruciform DNA structure with four arms by base pairing.^21^ The following year, Seeman's team^22^ designed cross-structured “four-armed junctions”, where each four-armed junction is referred to as a tile, and multiple tiles can be self-assembled into more complex 2D or 3D structures by adding sticky ends (Fig. 2a). However, the four-armed junction tile has a large flexibility of branching points due to its exposed sticky ends, which leads to poor stability of the assemblies, unpredictable shapes, and lower target yields. Seeman's team^23^ introduced the DNA double crossover (DX) model and designed the DX Tile consisting of two juxtaposed strands of double helix structures. The two DNA double helices are connected to each other by two different strand exchanges to form two crossovers. By crossing the structures between the double helix structures, the stability of the assembled structure is greatly optimized and the structure exhibits better rigidity. However, it still suffers from the problem of uncontrollable shape and size as well as the inherent structural stability of DX. Ye *et al.*^24^ constructed one-dimensional DNA nanotubes with a certain curvature DX Tile with five short DNA strands, and each DX was interlinked with each other by sticky ends, and one-dimensional DNA nanotubes with diameters in the range of 7–20 nm and lengths up to 50 μm were successfully constructed. In 2003, Yan's team^25^ combined the advantages of both four-armed junctions and DX by replacing the single double helix of each arm of the four-armed junction with a side-by-side double helix structure containing internal crosses (the DX structure), thus developing a more stable cross tile.

DNA self-assembly is a promising method for preparing nanostructures, and researchers have utilized carefully designed DNA sequences (interconnections between DNA strands) to build *e.g.*, multidirectional crossover (PX) tiles,^26^ multi-armed structures,^27^ multi-point stars,^28^ and periodic nanostructures.^29^ However, the flexibility of branched DNA structures makes it difficult to construct well-defined geometries, which has become a major challenge for DNA nanotechnology. Scientists have successfully prepared complex DNA nanostructures by skillfully designing rigid motifs and introducing flexible conformations, which have advanced the development of DNA nanotechnology. By using the tensile integral principle integration method, well-defined and complex nanostructures can be assembled in both 2D and 3D. The five-point star-shaped blocks (tiles) assembled from three DNA single strands were further assembled into structurally complex icosahedra after binding through the sticky ends between the blocks. DNA tiles undergo dramatic structural changes in the process, highlighting the advantages of conformational flexibility^30^ (Fig. 2b). In addition, a new method employs a polyhedral 12-arm linkage structure instead of the traditional sticky end hybridization, circumventing the lattice assembly challenges and temperature and salt concentration sensitivity issues triggered by the flexibility of the multi-arm structure. The method designs junctions that can act as branching points and connect different tiles, and utilizes an inverse hierarchical folding strategy so that the wires first self-assemble into monomers and then form branching junctions in the lattice, assembling the 12-arm structure into mono-dispersed cubo-octahedral (0D) and 1D, 2D, and 3D structural monolithic units, successfully and efficiently assembling complex DNA structures.^31^ These ordered nanostructures show a wide range of applications in various fields such as disease therapy and biosensing.

### 2.2. DNA origami

In 2006, Dr. Rothemund of Caltech debuted a new DNA origami technique.^32^ This technique uses the Watson–Crick base pairing principle to complementarily pair a single DNA strand of 7000 bp (M13 phage genomic DNA) with hundreds of short DNA fragments of 20–60 bp (known as ‘staples’). By a certain degree of folding and bending, two-dimensional symmetric nanopatterns with a diameter of 100 nm, a molecular weight of about 5000 kDa, and specific shapes, such as triangles, five-pointed stars, and smiley faces, can be constructed^32^ (Fig. 2c). Since the introduction of the DNA origami technique, the field has grown rapidly, with a variety of asymmetric two-dimensional patterns such as dolphins and Chinese maps.^33,34^

With the rapid development of DNA origami technology, its applications have also increasingly expanded. The length of the DNA scaffold plays an important role in the scale and complexity of DNA origami structures. How to expand the size of DNA origami has become a key challenge to promote the development of DNA origami technology. Currently, there are two strategy to expand the size of DNA origami: firstly, by lengthening or introducing new basic frameworks, such as using high temperature to convert double-stranded DNA to single-stranded DNA to make the strand lengthened, employing PCR to amplify the DNA scaffolds, and replacing M13mp18 with PHIx174 (*i.e.*, the introduction of phage DNA); and the second strategy involves the use of either blunt-end stacking or sticky-end hybridization. A well-known example in DNA origami is the flat square motif, which is formed by folding and stabilizing a long single-stranded DNA molecule into a defined shape through the binding of staple strands. To construct more regular and exquisite planar patterns, Jiang *et al.*^35^ developed a DNA origami planar tessellation method. By adjusting the interhelix parameter of the DNA origami tile conformation to 2.70 nm, they were able to produce high-precision planar tile patterns such as Platonic, Lavisian, and Archimedean. In addition, wireframe polyhedra with versatility can be constructed using the DNA origami technique. For example, Wei *et al.*^36^ successfully constructed complex ‘chimeric’ linear frameworks by innovatively combining linear DNA origami structures and ‘Lego’-style DNA modules. The introduction of hydrophobic interactions through Cho-DNA hybridization enabled the construction of diverse nanostructures on a single template, which opens up new possibilities for applications in molecular sensors and molecular machines. Drawing on the concept of children's origami, Do-Nyun Kim *et al.*^37^ implemented a reconfigurable and environmentally responsive DNA-wireframe paper structure by using DNA helical tubes as crease lines and edges, and by adding ‘glue strands’ for folding and ‘release strands’ for unfolding the wireframe paper structure.

Traditionally, building micron-sized DNA structures has often been limited by the size and shape of DNA origami. With the development of versatile submicron 3D technologies, the construction has become more efficient and easier. Fan *et al.*^38^ developed a sub-micron scale DNA self-assembly technique called ‘meta-DNA’ (M-DNA). The technology is based on a six-helix DNA origami bundle, with a dangling short single strand of DNA as the ‘m-base’ to build the basic unit. M-DNA inherits three key properties of traditional short ssDNA oligonucleotides: the ability to form stable bonds through complementary base pairs, excellent rigidity, and adjustable flexibility. Due to these properties, M-DNA is able to act as an enlarged version of DNA, enabling the construction of diverse self-assembled DNA structures, such as polyhedra and lattices, from submicron to micron scales (Fig. 2d). A recent study achieved dynamic expansion and contraction of DNA origami structures by inserting i-motifs or hairpin motifs at crossover regions, with the size tunable between 68 nm and 150 nm through pH adjustment or complementary strand addition. This ingenious design simultaneously addresses two key challenges in tumor drug delivery: the poor penetration and rapid clearance of small carriers due to high tumor interstitial fluid pressure, and the limited penetration capability of large carriers in dense tumor stroma despite their prolonged circulation and retention.^39^ Furthermore, Wei *et al.*^40^ realized a “cut-and-paste” process in DNA origami structures through multistrand displacement and hybridization steps, enabling topologically continuous transformations characterized by integer topological invariants. Notably, their 3D DNA origami was constructed by bonding corresponding helix ends on each side of a 2D origami, which not only provides superior spatial positioning advantages but also reduces unwanted interactions while enhancing assembly connectivity. Another study leveraged the addressability of DNA origami octahedral structures to orthogonally integrate different stimulus-responsive DNA hairpins at specific vertices, creating a dynamically reconfigurable DNA origami crystal system capable of precise multiphase transitions in response to external stimuli along both *XY*-axes and *Z*-axis.^41^ The system's dynamic transformation modes were integrated into a guidance pathway map, allowing customization of at least 60 distinct crystal transformation routes. This system serves as a universal platform for developing novel dynamic-responsive nanomaterials, establishing fundamental strategies for designing complex adaptive materials and pioneering new directions in dynamic functional material development.

In addition, some software programs such as caDNAno and CanDo are widely used for DNA origami structure design, which can assist in modeling and tailoring links to build nanostructures with a wide range of dimensions, including 2D and 3D.^42,43^ As an open-source software tool, caDNAno focuses on providing an intuitive design interface and an open architecture for plug-in creation. By utilizing caDNAno, the researchers were able to efficiently construct DNA nanostructures with customized shapes, such as squares, long bottomless tubes, 3D origami “rollers” and other complex 3D structures. CanDo, as an online tool, focuses on fast computational feedback and finite element analysis online. By predicting the 3D shape and mechanical flexibility of DNA origami nanostructures based on their sequence connectivity maps, CanDo successfully simulates the shape of complex 3D wireframe structures, including significant local buckling and deformation, demonstrating the predictive ability to handle complex structures.

### 2.3. Spherical nucleic acids

Mirkin *et al.*^44^ developed DNA sequence-modified gold nanoparticles (AuNPs) functionalized with alkyl sulfhydryl groups in 1996, which were able to form aggregates with stability despite high temperature and salinity conditions, and termed them SNAs. SNAs are 3D nanostructures composed of an inorganic or organic material as a core and conjugated to a regularly arranged and compact oligonucleotide shell^45^ (Fig. 2e). Cores are the basis for building SNAs and can be composed of AuNPs, silver nanoparticles (AgNPs), proteins, liposomes, or nucleic acids. The core supports the oligonucleotide shell, and the core size and shape affect the density of oligonucleotide coverage, with small cores and large radii of curvature promoting denser coverage and chain space. The shell of an SNA typically contains three key functional regions: (1) attachment ends for immobilization, (2) spacer regions, and (3) specific functional regions dedicated to the recognition of target genes. The recognition regions of oligonucleotides are modified by specific functional groups, such as the introduction of fluorophores, which can confer specific optical or electrical properties to SNAs. These material structures and coupling modes are often used in a variety of applications such as rapid detection of single nucleotide mutations, DNA array scanning detection, SNA scanning optics, and bio-barcoding technology, demonstrating SNAs as a well-characterized form of biomolecule detection, and laying the groundwork for the precise construction and functionalization of further nanomaterials.

SNAs have some unique advantages over other conventional nanostructures:^46^ (1) strong binding ability to complementary nucleic acids: the binding ability is affected by salt concentration, the length of the spacer sequence, and the oligonucleotide sequence; long strands and high salt concentration can enhance the binding, but an increase in the proportion of prehybridized strands will reduce the capturing ability. In addition, short internal complementary DNA strands can enhance SNA binding efficiency. (2) Low nuclease degradation: the spherical structure and dense oligonucleotide sequences on the surface of SNAs make them highly resistant to nuclease. Studies have shown that the half-life of SNAs is 4.3 times longer than that of free DNA, highlighting the importance of the high-density shell for SNAs’ anti-degradation ability. (3) Low incidence of immune response: SNAs are only one-tenth of liposomes, and the higher the density of oligonucleotides on their surface, the lower the probability of immune response, which may be associated with low IFN-β levels. (4) Cellular uptake rate of more than 99%: SNAs enter cells mainly through the endocytosis pathway mediated by SR-A and CAV-1. Based on SNAs, Mirkin and Distler's team^47,48^ developed DNA dendrites with high-density nucleic acid nanostructures for efficient delivery of biomolecules to living cells. It was shown that DNA dendrites enable rapid cellular uptake by precisely modulating the position of oligonucleotides on the surface of SNAs due to the highly oriented and densely packed arrangement of DNA branches, an effect attributed to their enhanced interaction with scavenger receptor class A (SR-A). In addition, the shape and structure of the oligonucleotide chains of the SNA shell can effectively modify cellular uptake. For example, Tian *et al.*^49^ constructed a DNA framework based on spherical nucleic acids (tDF-SNAs) through a self-assembly technique. The core diameter was 50 nm AuNPs, and approximately 82 tDFs decorated the surface of each particle in the form of tetrahedra. This structural design with greater freedom enhances interfacial interactions during DNA hybridization, which in turn enhances the efficiency of cellular uptake of tDF-SNAs (Fig. 2f). With the development of SNAs, the third generation of spherical nucleic acids, namely, supramolecular spherical nucleic acids (Supra-SNAs), was successfully developed. Chen *et al.*^50^ synthesized multisubstituted molecular spherical nucleic acids (m-SNAs) by click chemistry using β-cyclodextrin (β-CD) and adamantane (Ada) as templates. Accumulation of these Supra-SNAs in tumors was able to reduce HER2 protein expression, inhibit the PI3K–AKT signaling pathway, and enhance tumor cell apoptosis. Compared with single-stranded DNA, Supra-SNAs exhibit superior nucleic acid stability and cellular uptake, and can effectively promote apoptosis. At the same time, SNAs show great potential to be a flexible and biologically safe tool in the biomedical field due to their highly customizable and excellent biological properties with their diverse compositional options for both the nucleus and the shell.

### 2.4. DNA nanogels

DNA hydrogels (DNA nanogels) are 3D network gels that are formed by complementary pairing, cross-linking, or physical entanglement of DNA strands.^51^ There are structural and functional differences between DNA hydrogels and other nucleic acid-containing hydrogels. DNA molecules in DNA hydrogels not only assume the role of a cargo but also constitute the core of the 3D network structure for the hydrogel. DNA hydrogels show great potential for application in the biomedical field due to their unique dual properties.

There are currently two types of hydrogels which are constructed from DNA strands as units:^52^ pure/hybridized DNA hydrogels. Pure DNA hydrogels are synthesized physically or chemically using DNA strands and specific structural units (such as motifs). Common methods for purified DNA hydrogels include self-assembly of DNA structural units (*e.g.*, X-, T-, or Y-types), and rolled-circle amplification (RCA) using circular single-stranded DNA as a template. Liu *et al.*^53^ designed a dual-responsive DNA hydrogel (Fig. 2g) based on two modules, a Y-type scaffold and a linker, and hybridization of the Y-type scaffold to the sticky ends of a linear double-stranded DNA “linker”. RCA is an isothermal enzymatic amplification method capable of synthesizing extremely long DNA strands by mimicking the replication principle of circular DNA in nature. Yang's team^54^ utilized RCA technology to generate ultra-long DNA strands through the elongation and displacement of phi29 polymerase. Under oscillating conditions, these DNA strands are entangled with each other to form physical cross-linking points, constructing a 3D DNA hydrogel with ultramachine properties. This hydrogel behaves as a liquid when taken out of water, but possesses persistent solid-like properties in water. After functionalization, this hydrogel shows great potential in cell capture and release, disease diagnosis, and drug delivery (Fig. 2h). Multi-component/hybridized DNA hydrogels are DNA molecules bound to natural or synthetic polymers (*e.g.*, polyacrylamide) as well as other biomolecules through covalent or non-covalent interactions to enhance their mechanical strength, stability, and versatility. Matsuda *et al.*^55^ successfully prepared DNA–polymer hybrid hydrogels for the first time using DNA as a cross-linking agent in 1996. They polymerized acrylate short DNA chains with acrylamide monomers and formed DNA-PAA hydrogels by direct cross-linking or complementary pairing with DNA strands on the backbone. Later, Liao *et al.*^56^ proposed a strategy to stimulate responsive DNA-polyacrylamide assembled hydrogels to stabilize microcapsules. They coated the DNA with CaCO_3_ and subsequently crosslinked it with polyacrylamide chains to generate a DNA cross-linked hydrogel coating *via* the hybridization chain reaction (HCR). Deoxyribonuclease (DNAzyme) units are doped into the crosslinked hydrogel layer, and when the corresponding cofactor cleaves the crosslinked DNAzyme substrate, the hydrogel coating decreases in stiffness and increases in pore space, releasing the loadings. This DNA hydrogel is capable of stable loading of chemotherapeutic drugs by chemical bonding or physical adsorption, and by regulating the degradation rate of the DNA hydrogel, a sustained and controlled release of the drug can be achieved to optimize the effect of tumor therapy.

## 3. Drug delivery strategies for microenvironment-responsive DNA assemblies

The TME with properties such as weak acidity and abnormal expression of proteases makes DNA delivery systems unique in the field of cancer therapy. Different types of stimuli in the microenvironment, such as abnormal biological signals and pathological states, can trigger changes in the structure and properties of DNA assemblies. Generally, DNA assemblies are in the “off” state under normal physiological conditions and switch to the “on” state under specific conditions (*e.g.*, enzyme overdose), and are designed to precisely target the tumor site for drug delivery. These advancements have achieved great success in various cancer therapies (Table 1).

**Table 1:** Tumor microenvironment responsive DNA assembly drug delivery system

Stimulus	DNA structure	Drug classification	Disease	Cellular model	Animal model	Ref.
pH	DNA tetrahedron	Maytansine	Breast cancer	SKBR3, BT474, MCF-7 and MCF10A cells	SKBR3-tumor-bearing mice	57
pH	DNA–BSA nanocarrier	DOX	Cancer	HeLa cells	Balb/c nude mice	58
pH	CpG-MUC1-hydrogel/Dox	DOX + CpG	Breast cancer	MCF-7 and A549 cells	Breast cancer mouse models	59
pH	DNA hydrogel	Dox	Cancer	HeLa cells	—	60
pH	The origami robotic	Peptides	Breast cancer	SK-BR-3, HBEC-5i and HEK293 cells	SKBR3-tumor-bearing mice	61
pH	Spherical nucleic acid	Dox	Cancer	HeLa cells	—	62
MMPs	Spherical nucleic acid	—	Cancer	HT-1080 cells	Nu/nu mice	63
MMPs	Spherical nucleic acid	DOX + CpG	Cancer	HUVECs and E.G7-OVAs	C57BL/6 mice	64
TE	DNA network	—	Cancer	MCF-7 and BEAS-2B cells	Balb/c nude mice	65
TE	DNA icosahedron	Platinum	Cancer	U87MG cells	BCG823/DDP tumor-bearing nude mice	66
ATP	3D DNA nanostructure	DOX	Cancer	MCF-7 cells	Balb/c nude mice	67
ATP	SNAgel	DOX	Cancer	HeLa and HEK293 cells	Balb/c nude mice	68
ATP	DNA hydrogel	DOX	Cancer	MCF-7 and L929 cells	—	69
ROS	DNA hydrogel	DOX + RNase A	Cancer	MCF-7 and L929 cells	—	70
GSH	DNA tetrahedron	CPT	Cancer	HCT116 and MCF-7 cells	HCT116 tumor-bearing nude mice	71
GSH	DNA hydrogel	CPT	Cancer	HCT116 cells	Tumor xenograft resection mouse model	72
GSH	DNA tetrahedron	DOX	Multidrug resistant cancer	MCF-7 cells, MCF-7/R cells and SKOV3/R cells	SKOV3/R tumor-bearing mice	73
AS1411	The origami robotic	Thrombin	Breast cancer	HUVECs and MDA-MB-231 cells	C57BL/6J mice	74
AS1411	DNA tetrahedron	Melittin	Cancer	HUVECs and L929 cells	Human malignant melanoma xenograft mouse model	66
Folate	DNA nanotube	—	Nasopharyngeal epidermal carcinoma	Nasopharyngeal epidermal carcinoma KB cells	—	75

### 3.1. pH response

Due to the rapid proliferation characteristics of tumor cells, their nutritional supply becomes insufficient, causing a change in their energy metabolism mode. Most of the nutrients are converted to lactic acid and accumulate under the action of lactate dehydrogenase, resulting in a weakly acidic TME, known as the Warburg effect.^76,77^ Normal cells are usually neutral (pH 7.4) while tumor cells are weakly acidic (pH 6.5). This special cellular environment can be distinguished from normal tissues. Such special properties of the TME can be used to develop pH-responsive DNA histomes for drug delivery at tumor sites.

**Fig. 3:**
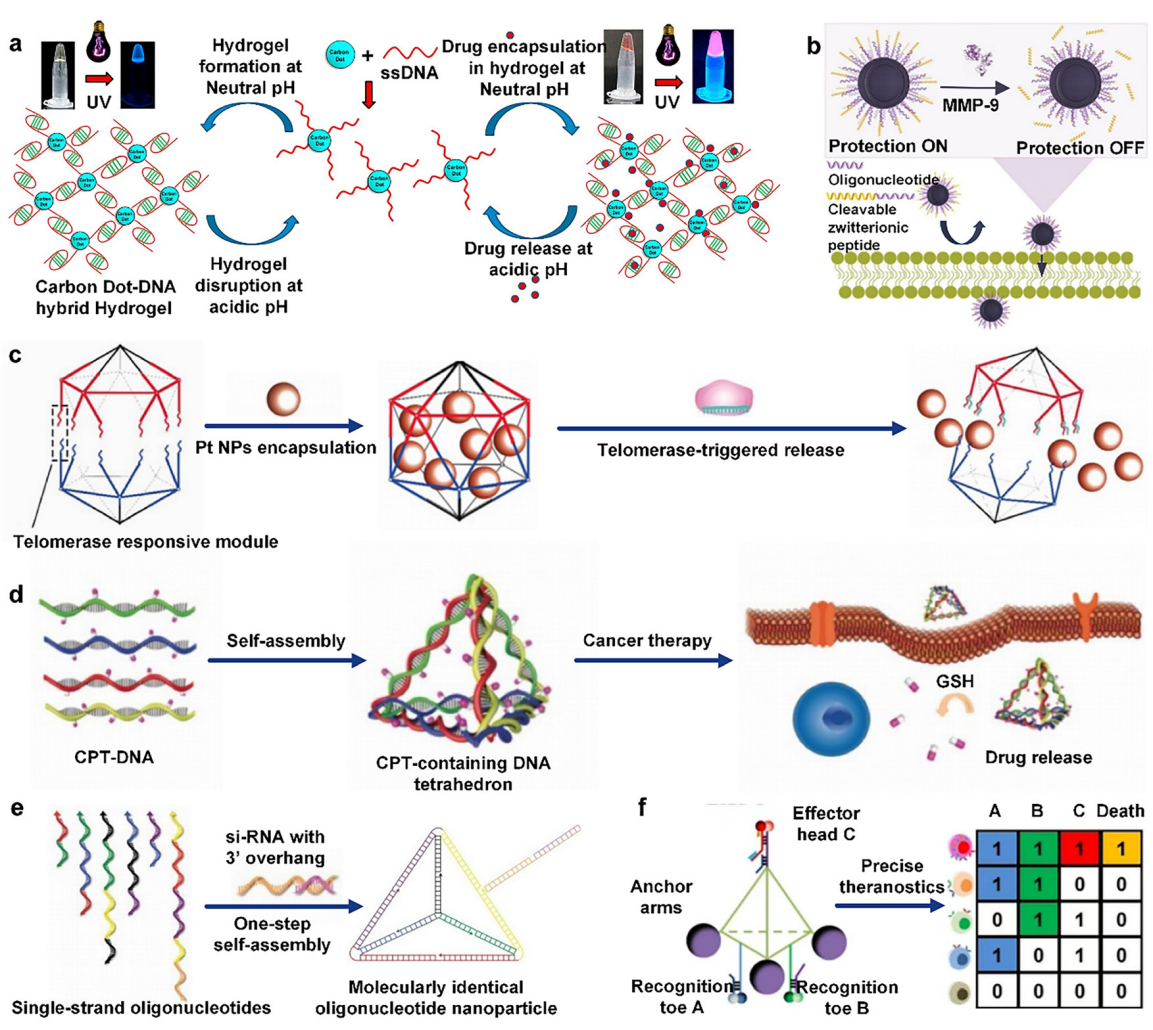
Drug delivery strategies for microenvironmentally responsive DNA assemblies. (a) Cytosine-rich DNA single strands are spliced with carbon dots to form hydrogels with i-motif sequences for adriamycin (Dox) delivery at tumor sites. Reproduced with permission from ref. 60. Copyright 2016, Elsevier Ltd. (b) Functionalized SNA in the presence of MMPs in the tumor microenvironment; the peptide coating is rapidly cleaved off to enhance tumor site uptake and accumulation. Reproduced with permission from ref. 63. Copyright 2022, American Chemical Society. (c) DNA icosahedra are disassembled by telomerase to achieve precisely targeted release of platinum drugs. Reproduced with permission from ref. 83. Copyright 2018, WILEY-VCH Verlag GmbH & Co. KGaA, Weinheim. (d) CPT complexed DNA tetrahedra respond to GSH in the tumor microenvironment for CPT-targeted release. Reproduced with permission from ref. 71. Copyright 2019, WILEY-VCH Verlag GmbH & Co. KGaA, Weinheim. (e) Folate ligand-modified DNA tetrahedra can deliver siRNAs to the tumor site smoothly and trigger gene silencing. Reproduced with permission from ref. 88. Copyright 2012, Springer Nature Limited. (f) Intelligent DNA logic-gated nanorobots for tumor cell killing. Reproduced with permission from ref. 89. Copyright 2021, WILEY-VCH GmbH.

DNA assemblies can undergo structural transformations in response to acidic microenvironments, a unique capability derived from their built-in responsive elements. Among these, pH-responsive DNA assemblies often employ the i-motif as the functional unit. The i-motif is a cytosine-rich quadruplex DNA structure where two parallel-stranded duplexes tightly intercalate with each other. Under acidic conditions, the cytosine residues become protonated to form hemiprotonated cytosine (C:C+) base pairs.^78^ Adjacent cytosines then interact through hydrogen bonding to create the core structural unit of the i-motif. This structural transition has been ingeniously engineered to serve as a crucial responsive module in DNA-based drug delivery vehicles. Liu *et al.*^79^ developed a DNA hydrogel assembled with a three-armed DNA structure (Y-unit) by intermolecular i-motif interactions under slightly acidic conditions. This DNA hydrogel was able to switch to a non-gelatinized state within tens of seconds when the pH was reduced or slightly acidic. In addition, researchers^80^ developed a supramolecular polymerization system based on a DX structure, which was achieved by designing a 44-mer single-stranded DNA (SDX) containing four self-complementary domains. Two of the longer self-complementary domains (16-mer each) were used for the formation of rigid DX monomers, while the two shorter self-complementary domains (6-mer each) were used for polymer extension. After the introduction of the half i-motif at the 3′ end of SDX, the hydrogels were crosslinked by interactions between the half-i-mers under acidic conditions to form an elastic hydrogel, which was able to respond to pH changes. Singh *et al.*^60^ developed a hydrogel based on functionalized carbon and cytosine-rich DNA with i-motif sequences, which solved the problems of low drug encapsulation porosity and limited efficiency of drug loading and delivery by electrostatically embedding adriamycin (DOX) into a network composed of carbon dots (CD) and DNA strands. It is stable at physiological pH, but is able to release DOX in the weakly acidic tumor microenvironment, effectively killing HeLa cells and showing potential in cancer therapy (Fig. 3a).

Dynamic DNA triplexes are formed through a combination of Watson–Crick base pairing and pH-sensitive Hoogsteen base pairing, and they can be formed after protonation under acidic conditions and dissociated under neutral or weakly basic conditions. Their sensitivity to pH changes can be increased by changing the relative content of the CGC/TAT triplex. Wang *et al.*^61^ developed a pH-responsive DNA origami robotic switching device that is capable of autonomously displaying cytotoxic ligand patterns in the tumor microenvironment. Under normal physiological conditions (pH 7.4), the device hides six ligands and maintains cellular inertia, whereas in an acidic environment (pH 6.5), the oligonucleotide strand (TFO) end binds to double-stranded DNA *via* Hoogsteen interactions to form triple-stranded DNA (tsDNA), which displays the ligands in a hexagonal pattern with a diameter of 10 nm, and is effective in aggregating death receptors and inducing apoptosis in human breast cancer cells. In addition, Li *et al.*^62^ designed a pH-responsive adriamycin-SNA conjugate by utilizing the structural tunability of SNAs and their ability to undergo rapid and reversible structural changes, and this SNA nanostructure was able to perform linear/ring stem switching *via* pH-sensitive parallel Hoogsteen interactions. Under low pH conditions, DOX molecules could be released from the SNA nanocarrier and were able to significantly reduce the cellular activity of the HeLa population.

### 3.2. Enzymatic response

The TME has abnormally high expression of multiple enzymes to promote proliferation, invasion, and metastasis of tumor cells. Enzyme-responsive stimulation is ideal for the biomedical field due to its strong biorecognition ability, high catalytic efficiency, warm reaction conditions, *etc.* DNA assemblies are able to effectively trigger enzyme reactions by precisely designing DNA sequences, thus achieving controlled release of drugs, which enables the distribution and accumulation of drugs at the tumor site and improves anti-tumor effects.

Matrix metalloproteinases (MMPs) are a family of zinc-containing proteolytic enzymes. In the tumor microenvironment, the overexpression of MMPs can serve as a specific biomarker for enzyme-responsive systems. MMPs primarily consist of two key domains: a zinc-containing catalytic domain and a hemopexin-like domain.^81^ The hemopexin-like domain is responsible for specific substrate recognition, while the zinc ion in the catalytic domain activates the enzyme and cleaves the substrate. In DNA-assembled drug delivery systems, this specific degradation enables the disassembly of particular substrates (such as SNAs) and subsequent drug release.^82^ Zhang *et al.*^63^ designed a novel SNA whose shell was encapsulated by an amphipathic peptide containing MMP recognition sequences. The amphipathic peptide shell avoids immune recognition of the SNA and prolongs its half-life in the circulation, while this design allows the SNA to shed its shell rapidly after activation by MMPs in the tumor microenvironment, which in turn promotes its accumulation and cellular uptake rate at the tumor site (Fig. 3b). Deng *et al.*^64^ modified a liposomal SNA using the DOPE–MMP-9–CpG self-assembling module, and this SNA was able to efficiently deliver DOX and CpG to the tumor site at the same time, and release them precisely under the action of MMP-9. In addition, this SNA was able to activate dendritic cells (DCs) and promote the proliferation and differentiation of CD8+ and CD4+ T cells, thereby effectively inhibiting tumor growth and prolonging the survival time of experimental animals.

Telomerase (TE) is a ribonucleoprotein enzyme that is active in most tumors and is essential for maintaining chromosome ends (telomeres). It is predominantly expressed in germ cells and stem cells in normal humans, whereas in tumor cells, TE activity is essential to support their unlimited proliferative capacity, making it an important target for anticancer therapy. Researchers^65^ developed a TE-mediated DNA self-assembler, which contains triphenylphosphine (TPP)-enabled Y-shaped DNA (TPP-Y-DNA) modules and TE-responsive junctional DNA (T-l-DNA). T-l-DNA contains both TP- and TE-recognized l-DNA. l-DNA is released from T-l-DNA in a TE-mediated strand displacement reaction after cancer cell uptake, and then assembles with Y-DNA to form DNA assemblies, which can effectively encapsulate and interfere with the normal function of mitochondria, and ultimately induce apoptosis of cancer cells. Ma *et al.*^83^ designed a TE-responsive DNA icosahedron consisting of two conical DNA cages linked by telomerase primers (TP) and telomeric repeat sequences. Platinum drugs were encapsulated within the DNA framework, and when TE was activated in cancer cells, primer extension triggered an intrastrand displacement reaction, which disassembled the cage structure and released the platinum drugs. The device achieves precisely targeted release of platinum drugs, which helps to reverse tumor resistance and reduce toxicity to normal tissues (Fig. 3c).

### 3.3. Endogenous small molecule responses

ATP, ROS, GSH and glucose serve as endogenous biostimulants that can interact with specific sequences in the DNA assembly to alter its structure, thereby enabling responses to biological signals at the molecular level.

Enhanced glycolytic processes in tumor cells result in much higher internal ATP concentrations (∼100 μmol) than in normal tissues and blood (∼10 μmol), and this shift in energy metabolism is a distinctive feature of malignant tumors.^84^ This property was utilized by Xu *et al.*^69^ to construct an ATP-responsive DNA hydrogel for DOX-targeted delivery. The system consisted of a polyacrylamide–DNA copolymer, an ATP aptamer, and DOX. When ATP is present, the hydrogel disassembles and releases DOX for the treatment of cancer cells. To enhance the release efficiency of chemotherapeutic drugs in specific cancer cells, Xiao *et al.*^68^ designed a DNA toe-modified spherical nucleic acid template hydrogel (SNAgel) as an engineered smart switch. This was achieved by covering the surface of AuNPs with a dense DNA shell and embedding the DNA toe switch. When ATP is present, the switch can be triggered to adjust the length of the toehold sequence, precisely controlling the kinetics of drug release and enabling rapid drug release. The compact DNA shell of the SNAgel enables it to prolong *in vivo* cycling time, precisely target tumors, promote intracellular uptake, and induce apoptosis.

The high level of ROS in cancer cells provides multiple ideas for designing intelligent ROS-responsive drug delivery systems. Zhang *et al.*^70^ developed a ROS-responsive DNA nano gel constructed from specific Y-shaped monomers and DNA junctions and encapsulated NBC-modified deoxyribonuclease I (DNase I). The system was able to encapsulate DOX and ribonuclease A (RNase A) as drug models in the nanogel by DNA embedding and electrostatic interactions, respectively. Under the high ROS environment in cancer cells, the NBC modification on the gel was removed, restoring its enzyme activity and prompting the nanogel to self-degrade and release the drug.

Cancer cells produce excessive ROS due to high metabolism, leading to oxidative stress. To defend against oxidative damage, cancer cells upregulate the expression of antioxidants such as GSH to neutralize ROS and protect themselves from oxidative damage. Some researchers have taken advantage of the property that disulfide bonds are stable in the extracellular environment of low GSH but are easily cleaved in the intracellular environment of high GSH, and used the disulfide bond-modified DNA assemblies as drug carriers to achieve specific release of drugs at the tumor site. Zhang *et al.*^71^ designed a stimuli-responsive drug-containing DNA framework as a delivery vehicle by conjugating camptothecin (CPT) to a polysorbate (PS)-modified DNA strand *via* carbon-ethyl bromide. The hydrophilicity of the DNA–CPT conjugate system can be improved by modulating the position of PS on the DNA strand. The disulfide bond inside the conjugate can trigger the release of CPT under the cleavage of a high concentration of GSH in the tumor microenvironment, which dramatically improves the efficiency of tumor therapy (Fig. 3d). Yang's team^72^ constructed DNA nanoframes (NFs) capable of loading Cas9 ribonucleoprotein and single-guide RNA to form RNA–DNA complexes through a cascade HCR initiated by acrylamide-modified DNA (acDNA). The high concentration of GSH in tumor cells was used to trigger disulfide bond breakage in the NF to achieve the precise release of Cas9 RNP.

Cancer cells consume large amounts of glucose due to rapid proliferation. Using glucose oxidase (GOx) can deplete glucose within the tumor and cut off its energy supply. Further integration of GOx into nanocatalysts can increase the concentration of H_2_O_2_ within the tumor and catalyze the generation of hydroxyl radicals *via* ferrocene (Fc) to effectively destroy tumor cells. This combined strategy can inhibit tumor growth more effectively.^85^ Zhao *et al.*^86^ developed a DNA hydrogel that integrated a GOx–Fc cascade reaction, which optimized the efficiency of the enzymatic cascade reaction by precisely controlling the distance between GOx and Fc, and most efficiently converted glucose into hydroxyl radicals to promote immunogenic cell death when the distance between them was 6.28 nm. In addition, this hydrogel can continuously release catalysts *in vivo* for more than 4 days, generating hydroxyl radicals for a long period and effectively destroying cancer cells.

### 3.4. Receptor targeted delivery

Overexpression of epidermal growth factor receptor (HER), a tyrosine kinase receptor that regulates cell growth, accelerates tumor cell growth, promotes angiogenesis, and enables invasive metastasis, and is common in malignant tumors such as non-small-cell lung, breast, and pancreatic cancers, and correlates with tumorigenesis, progression, and poor prognosis. Wu *et al.*^87^ designed the Nb-TET-56MESS conjugated DNA assembly, by cleverly embedding the platinum drug 56MESS into a double-bundled DNA tetrahedral structure to achieve efficient drug loading. This structure was able to precisely deliver the drug to HER1 overexpressing tumor cells by binding to the HER1 antibody, which enhanced the cellular uptake efficiency of the drug and effectively blocked HER1 signaling. Lin *et al.*^57^ developed a HER2-targeted aptamer-modified DNA tetrahedral delivery system, by combining the erythrocyte membrane and pH-responsive liposomes through bionic camouflage technology to enhance the *in vivo* retention time and the tumor aggregation effect of HER2-positive cancer therapeutics.

Overexpression of folate receptor alpha (FRα) in a variety of tumors makes it an important therapeutic target. Mao *et al.*^75^ embedded folic acid in DNA strands to create self-assembled micro-DNA nanotubes (DNA-NT). It was found that the functionalized DNA-NT entered the cells after folate-FRα-specific interactions and that the amount of folate on the DNA nanotubes was positively correlated with the uptake efficiency of nasopharyngeal carcinoma KB cells. Later, researchers^88^ utilized self-assembled DNA tetrahedra, which could deliver siRNA effectively, by precisely controlling their size and the distribution of surface ligands (folic acid). The results showed that folic acid-modified DNA tetrahedra were significantly better than unmodified controls in the gene silencing effect (Fig. 3e).

In the absence of tumor-specific antigens, single or multiple biomarker recognition may lead to non-specific binding and false positives, triggering unnecessary drug internalization. The diversity of receptors on the surface of eukaryotic cells results in a single-receptor recognition system that is prone to off-target toxicity, whereas multi-receptor analysis allows for more precise identification of disease-specific cells, improving diagnostic and therapeutic accuracy. The secondary DNA logic gated nanorobot (DLGN) is a precision medical tool consisting of an aptamer, an anchoring robotic arm, and a drug-coupled aptamer (paclitaxel and microtubule protein coupling) that recognizes and anchors to live cells expressing specific markers. When the target cells highly express the three markers, mucin-1 (MUC1), epithelial cell adhesion molecule (EpCAM), and protein tyrosine kinase 7 (PTK7), the secondary logic gate of the DLGN is activated, releasing the drug for precise diagnosis and treatment of the target cells. This technique improves the precision and efficiency of treatment by creating barcodes for each cell through a unique fluorescence pattern^89^ (Fig. 3f). In addition, triple receptor-targeted therapy is an advanced therapeutic strategy that enhances therapeutic efficacy by simultaneously targeting three different receptors or targets. The smart-responsive DNA nanoscale precision-guided missile (D-PGM) is composed of a warhead (WH), which is formed by four DNA strands creating a wire skeleton, and a guidance/control (GC), which is constituted by four DNA strands forming a logic circuit for targeting cancer cells. D-PGM utilizes the AND logic gate mechanism in GC to achieve precise drug delivery by assembling aptamers TC01, Sgc4f, and Sgc8 to form a three-dimensional structure that specifically binds to tumor cell surface receptors. The GC system probe components are labeled by different fluorophores, enabling real-time monitoring of GC disassembly, cell internalization, nanocarrier distribution, and changes in the relative position of the drug with respect to the carrier during drug delivery.^90^

## 4. Biosensing and imaging function for switchable DNA assemblies

Based on the editable nature of DNA, DNA sequences can be precisely designed and synthesized for biomedical applications. The applications of DNA assemblies in biomedical fields include the following: (1) biosensing: DNA assemblies are capable of converting the presence of biomarkers into detectable signals, such as electrochemical or fluorescence signals, thus enabling the detection of a wide range of biomarkers; (2) bioimaging: by visualizing DNA assemblies, it is possible to track the location and concentration of biomolecules (*e.g.*, RNA, enzymes) within a cell, helping to study their dynamics within the cell.

### 4.1. DNA assemblies for biosensing

DNA nanotechnology has great potential in biosensing. By precisely controlling the size and structure of DNA assemblies and combining them with functional modules, it is possible to develop sensors that are both specific and highly sensitive. In general, sensors consist of a recognition unit and a sensing unit. The recognition unit is responsible for detecting the target, while the sensing unit converts the intermolecular interactions into measurable physical signals (*e.g.*, optical and electrochemical signals, *etc.*) to detect changes in various physicochemical parameters within the cell. Typically, there are two output modes for assay results: using changes in the optical signal to identify the chemiluminescence of a biomolecule or using changes in the electrical signal to detect the electrochemiluminescence (ECL) of a biomolecule. This section will focus on these two output modes and describe how researchers can use these tools to detect and analyze various types of biomolecules.

#### 4.1.1. Fluorescence sensing

Fluorescent biosensors are an important signal output method based on DNA assemblies, the core of which lies in the use of fluorophores covalently or non-covalently bound to DNA assemblies to achieve biological detection of target molecules. Three main detection strategies have been used for DNA assembly-derived fluorescent sensors:^91^ (1) direct fluorescence enhancement, *i.e.* direct fluorescence emission; (2) FRET, involving the transfer of energy between two fluorophores; and (3) fluorescence burst, *i.e.*, the diminution or disappearance of fluorescence intensity.

Direct fluorescence enhancement refers to the emission of photons when a fluorescent molecule absorbs excitation light and returns directly from the excited state to the ground state, without involving other energy transfer or conversion mechanisms. Chen *et al.*^92^ prepared a periodic DNA nanoribbon (DNR-T) sensor with a structure consisting of a long DNA single strand generated by rolled circular amplification (RCA) and three short 32 nt DNA strands folded. They integrated the pH-sensitive carboxyfluorescein (FAM) into DNR-T and utilized its periodic structure and rigidity characteristics to effectively avoid the fluorescence burst problem caused by dye aggregation. The detection of intracellular pH is realized by ratiometric fluorescence changes.

The sensors that are based on DNA origami technology are able to detect intermolecular proximity using FRET techniques because when donor and acceptor fluorophores are close enough (<10 nm), the donor can non-radiatively transfer excitation energy to the acceptor, resulting in acceptor fluorescence emission. Ding *et al.*^93^ developed a triangular DNA origami-based FRET sensor using ATTO542 and ATTO647N dyes as donors and acceptors labeled on the DNA origami arms. Without the target, the dye hybridizes through 8 base pairs of DNA, resulting in a closed structure in a parallel arrangement, and the distance between the fluorophores is short and the FRET efficiency is high; upon target presentation, the DNA structure opens at an angle of 90°, and the fluorophore distance increases and the FRET efficiency decreases. The sensors mimic DNA strand substitution reactions to effectively detect different conformational changes and achieve high contrast optical detection of target molecules. Changes in the pH of the solution can induce alterations in the fluorescence properties of the dye pair, resulting in changes in FRET efficiency, which subsequently affects the fluorescence of the target molecule. Light intensity enables the sensor to sense environmental pH changes. A pH-sensitive fluorescent sensor based on DNA origami structures uses Cy5 and Cy3 as donor and acceptor dyes. These two dyes are labeled at both ends of a specific DNA structure and hybridized into a microscaffold. When tsDNA is activated, bringing Cy5 and Cy3 closer together enhances the FRET signal. In breast cancer cells (pH < 6.8), FRET occurs and tsDNA is turned on, while in normal tissues or cells (pH 7.4), FRET disappears and tsDNA is turned off.^61^ It has been shown that the arrangement of dyes, including their position and number, is a key factor in determining the sensitivity of the complex.

**Fig. 4:**
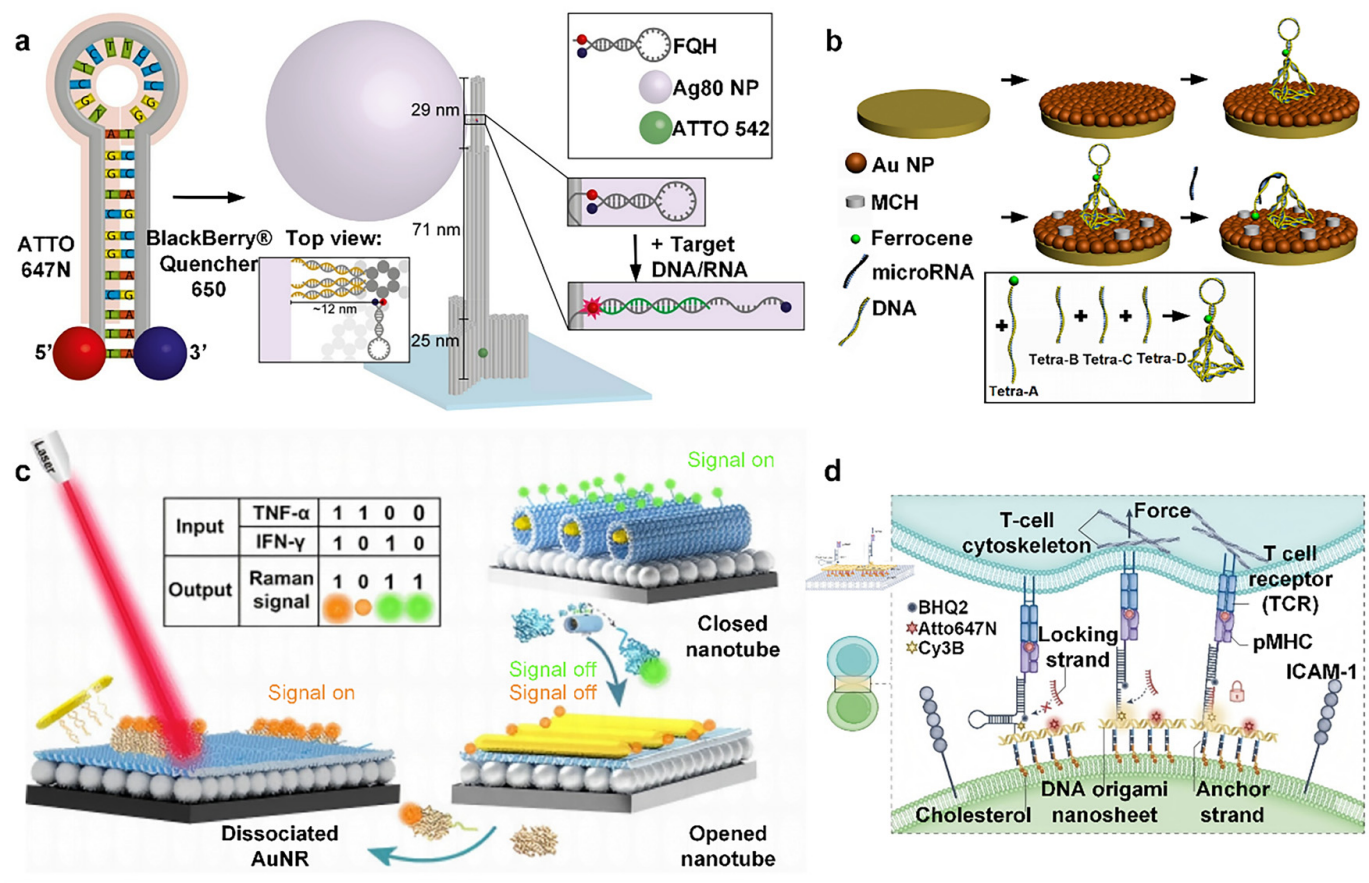
DNA assemblies in biomedical assays. (a) Fluorescence quenching hairpin (FQH) was integrated into DNA nanocolumns to make the dye luminescent by adding specific target DNA. Reproduced from ref. 94 with permission of the Creative Commons CC-BY. (b) AuNPs were deposited on a gold electrode, and 3D DNA probes self-assembled from four oligonucleotides were constructed for microRNA detection. Reproduced with permission from ref. 96. Copyright 2015, Elsevier B.V. (c) DNA origami plasma nanotubes, complexed with AgNP/Si, modulate their Boolean logic gates by reading TNF-α and IFN-γ and output Raman signals for cytokine monitoring. Reproduced with permission from ref. 101. Copyright 2024, American Chemical Society. (d) DOTS is a DNA hairpin-based force-sensing tool composed of TCR-targeting pMHC ligands and FRET pairs, which is anchored to the cell membrane *via* DNA nanosheets. When mechanical force is applied to the TCR, the hairpin structure extends, causing separation of the FRET pair and resulting in detectable fluorescence changes. Reproduced with permission from ref. 102. Copyright 2024, Springer Nature Limited.

A fluorescence burst is a phenomenon in which fluorescent molecules experience a decrease in fluorescence intensity when they encounter certain substances (bursting agents). Researchers^94^ designed a fluorescence-quashed hairpin (FQH) probe with a 12-base-pair stem and a 12-nucleotide loop, labeled with the ATTO 647N dye at the 5′ end and paired with the BBQ-650 quencher at the 3′ end to form a static dye–quencher pair. The probe was integrated into a DNA origami structure and immobilized on a coverslip by biotin. In the presence of target DNA, the probe structure opens, separating the fluorophore and the quencher, emitting red fluorescence; in the absence of bound target DNA, the fluorescence is quenched and barely glows (Fig. 4a). Ke *et al.*^95^ described a DNA origami-based nano-actuator capable of changing shape in response to effector binding. The actuator consists of four arms that form a diamond-shaped structure, and its conformational change can be controlled in two ways: either by “linking pillars”, adding two rigid DNA double helices, or by “locking corners”, which is regulated by the DNA strand alone. When corner-locking chains are present, changes in one half of the rhombus are accurately transmitted to the other half, achieving a substantial movement that results in a change in the fluorescence signal of the 6-FAM/BHQ-1 fluorophore burst pair.

#### 4.1.2. ECL-based biosensing

ECL detectors collect the generated light radiation to obtain ECL signals. ECL combines the advantages of electrochemical detection and chemiluminescence and has attracted much attention for its high sensitivity, strong selectivity, and wide linear range. The unique physicochemical properties of nanomaterials are often applied to construct novel biosensors with high sensitivity and stability. In particular, DNA nanostructures, due to their good biocompatibility and hydrophilicity, have less specific adsorption on the electrode surface, can monitor biomolecules directly inside the cell in real-time, and have been widely used in the field of detecting multi-targets such as nucleic acids, proteins, enzymes, and small molecules.

Currently, the main challenge faced by nucleic acid testing in clinical applications is its low content in clinical samples, and early detection of tumors relies mainly on blood biomarkers (*e.g.*, nucleic acids, proteins, *etc.*). However, these biomarkers are heavily diluted by the blood after they are detached from the tumor and enter the blood circulation, making their content low and the specificity of the assay consequently reduced. In contrast, DNA assemblies have the ability to carry more electrically active molecules, allowing effective electrochemical signal amplification through catalytic electrode reactions.

The development of lung cancer is often accompanied by the abnormal expression of microRNAs. Therefore, the highly sensitive detection of lung cancer-specific microRNAs provides an effective strategy for the early diagnosis of lung cancer. Liu *et al.*^96^ designed a 3D DNA origami-based structure, which consists of stem-loop structured DNA with Fc labeling and sulfhydrylated tetrahedral DNA nanostructures at the bottom. First, AuNPs were electrochemically deposited on a bare gold electrode, and then 3D DNA probes consisting of four oligonucleotides were constructed and allowed to self-assemble on the electrode surface. Next, the AuNP-coated surface was sealed using methyl cellulose (MCH) to prevent nonspecific adsorption. When the target microRNA is present, it opens the stem-loop structure of the 3D DNA probe, allowing the Fc group to contact the electrode surface, resulting in a detectable electrochemical signal (Fig. 4b). In addition, researchers^97^ designed a DNA hydrogel biosensor. They combined acrylate-DNA chain polymers with ferrocene recognition probes and allowed them to self-assemble and form a hybrid DNA hydrogel. Then, the hydrogel was immobilized on a silanization-treated ITO electrode. When the target miR-21 is present, the recognition probe hybridizes with it to form a double helix structure and releases it from the surface of the ITO electrode, which enables effective detection of miR-21 for early diagnosis of lung cancer.

Researchers^98^ designed a paper-based ECL origami device incorporating RCA technology and oligonucleotide-functionalized carbon dots to achieve ultrasensitive detection of IgG antigens. The device employs 3D macroporous gold paper electrodes to capture antibodies, while secondary antibodies on gold nanoparticles bind to the primer strand to form single-stranded oligonucleotides that act as primers for the RCA reaction. Catalyzed by the nucleotides and phi 29 DNA polymerase, the RCA reaction generates micrometer-length single-stranded DNA. These RCA products act as templates for the assembly of carbon dots (CDs), which facilitates the linear cyclic assembly of a large number of complementary DNA detection probes, resulting in significant enhancement of the detection signal. With the elevated ECL intensity of the cascade DNA nanolabel, the device demonstrated a wide detection range and excellent sensitivity using human IgG as a model.

#### 4.1.3. Plasma sensing

In addition to fluorescence and electrochemical sensing, there are several reports on the utilization of modulation changes in plasma as a sensing signal. Researchers^99^ developed a surface plasmon resonance (SPR) biosensor based on DNA origami and shearing techniques for the ultrasensitive detection of circulating tumor DNA (ctDNA) associated with lung cancer. They anchored three sets of DNA single strands to the gold-plated sensing domains, and subsequently the poly(A)-modified DNA strands were able to self-assemble into DNA tetrahedra at suitable ambient temperatures after binding to AuNPs. The DNA tetrahedral probes were able to be uniformly distributed in the gold-plated sensing region, maintaining the precise distances between individual AuNPs and the substrate and AuNPs. When the target DNA is added, DNA scissors cleave the DNA single strand within the DNA tetrahedron, releasing the AuNPs, and resulting in a decrease in surface loading and attenuation of the SPR signal. The researchers applied this sensor to clinical samples, some of which contained mutations in the EGFR and KRAS genes, and the SPR biosensor was able to achieve precise identification of the T790M mutation in the EGFR gene and the G12C mutation in the KRAS gene in patients with non-small cell lung cancer. Ding *et al.*^100^ developed a label-free, real-time surface plasmon resonance SPR biosensor based on hydrogel-gold nanoparticle supramolecular spheres (H-Au) for the detection of prostate cancer cell-derived exosomes. The sensor combines the signal amplification effect of hydrogels, the localized surface plasmon resonance (LSPR) effect of AuNPs, and a highly specific aptamer to achieve a wide linear range and low detection limit of total prostate-specific antigen (t-PSA), which shows good utility in human serum and has a great potential for application in disease diagnosis and bioanalysis.

In addition, the researchers designed a surface-enhanced Raman scattering (SERS) assay based on DNA origami plasma nano-antennas for monitoring cytokines relevant to cancer immunotherapy, such as tumor necrosis factor-α (TNF-α) and IFN-γ. These nano-antennas consist of self-assembled DNA nanotubes containing miniature gold nanorods (AuNRs) inside and attached to AgNP modified silicon wafers. TNF-α and IFN-γ are able to logically modulate the switch of the nanotubes, dissociating the AuNRs from the DNA structure. Taking advantage of this property, the researchers constructed Boolean logic gates with cytokines as inputs and Raman signal changes as outputs. The system was able to quantify TNF-α and IFN-γ in the serum of tumor-bearing mice receiving different immunotherapies, demonstrating the potential of DNA origami for the detection of a wide range of cytokines in a practical system^101^ (Fig. 4c).

#### 4.1.4. Biosensing DNA assembly in variable regions of cells

In recent years, the integration of DNA nanotechnology and biotechnology has driven the development of sensors based on programmable DNA assemblies for cell membranes and intercellular applications. This convergence has not only spurred innovation in related technologies but also provided new tools and methods for researching cell membrane and intercellular sensing. For instance, Zeng *et al.*^103^ utilized the spatial positioning benefits of framework nucleic acids (FNAs) in developing a 3D-DNA nanocage with the ability to dynamically anchor and sense on the surfaces of individual living cells. This probe, conjugated with cholesterol (chol)-labeled ssDNA, rapidly anchored to the cell surface within a short time. Compared to the control group without chol labeling, the chol-labeled 3D-DNA nanocage exhibited significant FAM signals. Furthermore, DNA assemblies have the ability to serve as bridging units between cells, accurately modulating intercellular interactions by efficiently anchoring to cell membranes and regulating spatial arrangement and functional modifications with precision. For example, Li *et al.*^104^ developed an efficient membrane-anchored nanoplatform based on programmable amphiphilic tetrahedral DNA nanostructures (TDNs), self-assembled from four DNA strands (S1, S2, S3, and S4). One vertex has a dangling DNA probe, while the remaining vertices are labeled with cholesterol tags for stable self-assembly and anchoring to the cell membrane *via* tunable hydrophobic vertices. The three-dimensional structure significantly enhances target-binding efficiency. To investigate the impact of intercellular contact zone size on cell function, Lin *et al.*^105^ further designed a contractile DNA nanoscale joint (DNJ). By using membrane-anchored TDNs of varying sizes, the membrane spacing at the antigen presenting cell (APC) interface could be extended, maintained, or shortened. The membrane spacing at the APC–T cell interface was modified to 10 nanometers in experiments, imitating the regulation of membrane spacing around T cell receptor–peptide-major histocompatibility complex (TCR–pMHC) in an authentic dendritic cell (DC)–T cell interaction system.

However, cells are constrained by various physiological factors, making this single membrane-anchoring strategy insufficient to reflect the dynamic biological processes of living cells. Dynamic alterations in the cell membrane structure are commonly facilitated by the interactions of lipids and proteins. Leveraging this property, researchers have explored the biosensing applications of DNA–protein and DNA–lipid hybrid assemblies in membrane-related studies. For example, You *et al.*^106^ designed a DNA probe inspired by miniature motor proteins in cells. They utilized the live cell membrane and its components as tracks and anchoring sites to construct a dynamic DNA system. Probe translocation between anchoring points was achieved *via* DNA strand displacement reactions, enabling the measurement of encounter kinetics between membrane components. Additionally, mechanical forces represent a key focus in biosensing research. Cellular traction forces, which are mechanical forces exerted by cells on their microenvironment, impact the majority of cells within intricate mechanical circumstances. Accordingly, researchers have developed programmable membrane force sensors based on DNA assemblies to precisely measure mechanical signals on cell membranes. These sensors typically consist of four components: (1) a ligand binding to membrane receptors or cholesterol, (2) a force-sensitive element (*e.g.*, a stem-loop hairpin structure) serving as a “spring”, (3) a fluorescence reporter system (*e.g.*, FRET fluorophore pairs, fluorophore–quencher pairs, or single fluorophores) acting as a “ruler”, and (4) a substrate-binding site for sensor immobilization. When cellular forces act on the DNA assembly, the force-sensitive element undergoes structural changes such as stretching, twisting, or breaking, altering the state of the fluorescent reporter. By monitoring fluorescence intensity changes, high-sensitivity mechanical force sensing can be achieved. For instance, Rémi Merindol *et al.*^107^ developed a stretchable DNA hydrogel film that adheres to the cell membrane surface, quantifying mechanical forces based on the breakage extent of FRET-labeled DNA strands. Meanwhile, Hu *et al.*^102,108^ designed a DNA origami-based tension sensor (DOTS) to measure forces exerted by T-cell receptors (TCRs) at fluid interfaces. DOTS consists of a force-sensitive DNA hairpin, a pMHC ligand targeting TCR, and a FRET pair, anchored onto a DNA origami nanosheet embedded in the cell membrane. When mechanical force acts on the TCR, the hairpin structure extends, separating the FRET pair and inducing fluorescence changes. Additionally, a complementary DNA “lock strand” captures transient TCR forces. This design combines the precision of DNA origami with the mechanical sensitivity of DNA hairpins, utilizing FRET signal analysis to construct a sensor platform for mechanical force measurement (Fig. 4d).

Apart from acting as frameworks for direct binding, probing, and editing biological membranes, dynamic DNA assemblies can also be used to detect various parameters (such as pH, ions, metabolites, *etc.*) in living cells and organisms. Leveraging the synthetic nature of DNA scaffolds, the incorporation of organic fluorophores enables protein localization tracking and dual-color readouts for reporting ionic environments. For instance, Modi *et al.*'s SimpHony strategy utilizes a FRET-based DNA nanomachine to map analytes like pH at the same time.^109^ This strategy utilizes molecular programming to position nanomachines along distinct pathways, allowing concurrent mapping of pH regulation in two different endocytic pathways within the same live cell. Additionally, capitalizing on the elevated extracellular H^+^ and K^+^ levels in tumor cells, cell-surface-anchored DNA computing systems can enhance tumor-targeted drug delivery and precision therapeutic efficacy. Li *et al.*^110^ developed an FNA-based logic-gated nanoplatform using *in situ* dimerized FNAs on cell surfaces for real-time monitoring of extracellular H^+^ and K^+^ in tumor cells, thereby improving the programmability and efficacy of precision therapy. This platform integrates a pH-responsive i-motif and a K^+^-responsive bimolecular G-quadruplex (bi-G4) to construct a novel DNA unit (tb-G4), which exhibits synergistic responsiveness to H^+^ and K^+^. The assembled heterodimeric structure enables specific binding, and through FRET signal analysis and cell-surface quantification, it achieves *in situ* sensing of the tumor microenvironment.

### 4.2. DNA assemblies for biomedical imaging

DNA assemblies are ideal materials for biomedical imaging due to their low immunogenicity. DNA assemblies can carry multifunctional units to enhance the sensitivity and specificity of imaging, such as fluorophores, signal amplification elements, and targeting ligands, which are valuable for personalized medicine and precision imaging.

**Fig. 5:**
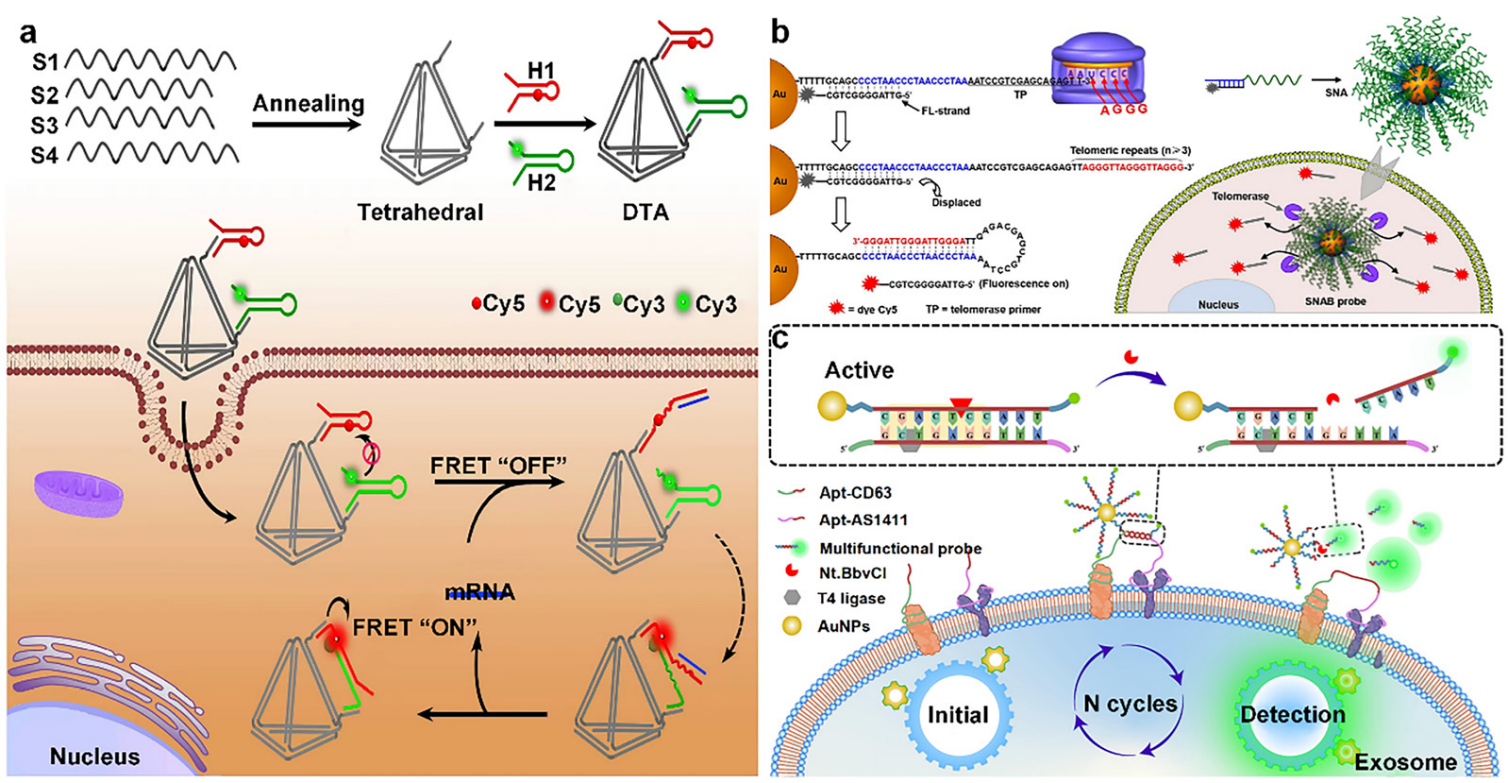
DNA assemblies in biomedical imaging. (a) DNA tetrahedra were assembled with two DNA hairpins and mRNA was detected by FRET signaling changes. Reproduced with permission from ref. 112. Copyright 2019, Elsevier B.V. (b) TP and FL strand were immobilized on the surface of AuNPs as the core to form SNAs, and telomerase activity was detected by the change of fluorescence signal. Reproduced with permission from ref. 119. Copyright 2018, American Chemical Society. (c) SSNAs utilize AuNPs as a core to construct nanoprobes to detect glioma-derived exosomes by fluorescence signal changes. Reproduced with permission from ref. 120. Copyright 2024, Elsevier B.V.

Non-invasive *in situ* imaging technology is different from traditional tumor detection means, avoiding possible invasive manipulation, and directly detecting and imaging tumors based on intracellular non-amplified RNA, an operation that maximally restores the true state of intracellular RNA. Wan *et al.*^111^ developed a DNA tetrahedron of T-TED for monitoring the dynamics of mRNA in living cells. T-TED consists of four DNA strands forming two triangular structures and a flexible hinge. In the absence of target mRNA, the donor–acceptor pairs were separated due to electrostatic and spatial repulsion. When target mRNA was present, the hybridization induced a conformational change in the T-TED probe, transforming it from a 2D to a 3D structure, which enabled efficient energy transfer from the donor–acceptor pairs. Single-molecule RNA fluorescence *in situ* hybridization enables the study of the abundance, distribution, and mobility of endogenous mRNAs in living cells. To enhance the detection sensitivity of DNA assemblies, researchers have developed a variety of enzyme-free signal amplification techniques that are capable of intracellular signal amplification and have been shown to be effective for DNA nanostructure-mediated intracellular imaging analysis. In recent years, non-enzymatic signal amplification strategies, including CHA and HCR, have developed rapidly. Combining non-enzymatic signal amplification strategies with DNA assemblies enables *in situ* imaging of intracellular RNA. CHA is an enzyme-free signal amplification technique for measuring the concentration and distribution of biomolecules in DNA structures. He *et al.*^112^ developed a DNA tetrahedral amplifier (DTA) for efficient imaging of mRNA in living cells. DTA consists of a tetrahedron of four DNA strands and two specific substable DNA hairpins (H1 and H2). In the presence of the target mRNA, DTA triggers the CHA reaction to form the H1–H2 double-stranded structure, which generates the FRET signal for amplified mRNA detection (Fig. 5a). Nucleic acid circuits demonstrate considerable potential for the amplification detection of biomarkers of interest in biologically significant engineering applications. Wang *et al.*^113^ developed a high-sensitivity nucleic acid detection system without enzymes by combining two signal amplification techniques, CHA and HCR, which are important for biomarker detection. The circuit catalyzes the target into a double-stranded DNA product, triggering the autonomous activation of the HCR hairpin, formation of HCR copolymer nanowires, and generation of a FRET signal. The optimized CHA–HCR system can cascade amplifiers suitable for the detection of low-expression endogenous analytes. In addition to nucleic acid, DNA assemblies are also being used for the detection of other biomolecules. As a major intracellular energy carrier, ATP plays an important regulatory role in a variety of biological processes. Currently, ATP imaging mainly relies on optical fluorescence detection techniques, but ATP aptamers may be activated during delivery and internalization, which can interfere with the *in situ* imaging of ATP. Yang *et al.*^114^ developed a DNA tetrahedron-based assembly for the ultrasensitive detection of small intracellular molecules such as ATP. The machine contains three modules: an aptamer for target recognition, an entropy-driven unit for a signal reporter, and a tetrahedral oligonucleotide for transport. When ATP binds to an aptamer, it triggers signal amplification and output. The nanomachines can also be fed into living cells to enable intracellular ATP imaging. Liu *et al.*^115^ constructed a reductase and light-responsive nanodevice for spatiotemporally controlled monitoring of biomolecules in subcellular organelles under hypoxic conditions. Using mitochondrial ATP as a monitoring target, the Y-shaped nucleic acid aptamer (Y-apt) in the device displays a “FRET ON” signal *via* FRET in the absence of light, and is activated by light and becomes ATP-sensitive, resulting in “FRET OFF”. The Y-apt involves FRET in the absence of light. When activated by light, it becomes sensitive to mitochondrial ATP, resulting in “FRET OFF”. When the nanodevices enter hypoxic cancer cells, they are degraded by reductase enzymes, releasing Y-apt, which in turn responds to mitochondrial ATP and emits fluorescence signals. This technique enables precise control of the fluorescence-sensing activity of ATP and spatial and temporal control of its activity by light. Intracellular ions play a key role in cellular metabolism and signaling, and dysregulation of ion homeostasis is associated with a wide range of diseases, so detecting changes in intracellular ions can help diagnose the early development of related diseases. Chen *et al.*^116^ designed a luminescent assembly based on DNA tetra- and triple-stranded bodies for monitoring K^+^ and pH in the lysosomal lumen, respectively. The assembly consists of upconverting nanoparticle luminophores and gold nanoparticle quenchers capable of generating green and blue luminescence signals for K^+^ and H^+^, respectively. By co-imaging in cells with both sensors, we discovered that H^+^ influx is correlated with K^+^ efflux. TE is an alkaline nucleoprotein reverse transcriptase complex that is activated to complement telomere length in cancer cells and thus is always highly expressed. This feature allows TE to be used as an early biomarker for cancer surveillance. Meng *et al.*^117^ developed a novel structurally switchable tetrahedral DNA nanoprobe (TDNp) specifically for imaging TE in living cells. This TDNp consists of three key components: a DNA tetrahedral structure, a TP, and a molecular beacon. When TE is present, it prompts the primer to extend, which in turn releases the molecular beacon and triggers FRET signaling. Therefore, TDNp can effectively distinguish between cancer cells and normal cells and monitor changes in TE activity in real-time. This technology provides a stable and reliable imaging tool for early diagnosis of cancer. Lin *et al.*^118^ developed a smart DNA hydrogel for targeted imaging of cancer. This hydrogel consists of Y-shaped DNA units and DNA junctions with fluorescence/quenching markers. When TE is overexpressed in tumor cells, the nano-hydrogel collapses to restore the fluorescence signal, enabling detection and imaging of TE activity. Liu *et al.*^119^ developed a novel SNA and SNA beacon combination technique for the visualization and detection of TE activity in living cells and animal models. This technique utilizes the cell-penetrating ability of SNAs to immobilize molecular beacons on the surface of AuNPs. Molecular beacons contain a telomerase primer TP and a fluorescently labeled DNA strand (FL strand); in the presence of telomerase, the TP strand is elongated, the FL strand is released, and fluorescence is restored, enabling the detection of telomerase activity. This technique provides a new approach to cancer diagnosis and treatment (Fig. 5b). Tumor-derived exosomes contain a variety of proteins derived from cancer cells and are one of the biomarkers for cancer diagnosis. In the field of cancer diagnostics, direct and accurate analysis of low concentrations of tumor-derived exosomes in complex biological samples is essential for non-invasive cancer diagnosis. Xu *et al.*^120^ proposed a new strategy based on super-spherical nucleic acids (SSNAs) coupled with cleaving enzyme-triggered signal amplification for the direct detection of exosomes of glioma origin. The SSNAs utilized AuNPs as a carrier carrying a multifunctional DNA probe, and the exosomes were labeled with two aptamers. When SSNAs recognize the labeled exosomes, they trigger relative gear movements, which act as exosome-specific retrieval on the one hand and are consumed as fuel on the other hand, outputting high-gain fluorescence signals. This strategy has excellent specificity and ultra-high sensitivity, providing new possibilities for early diagnosis and grading analysis of gliomas (Fig. 5c).

## 5. Conclusions

In summary, programmable DNA assemblies demonstrate tremendous potential in tumor therapeutic drug delivery, biosensing, and imaging applications. In tumor drug delivery, DNA assemblies can be designed with specific sequences to respond to endogenous biomolecules in the tumor microenvironment (such as acidity, enzymatic activity, ATP, GSH, and glucose). Through responsive structural isomerization, they enable rapid drug release while overcoming challenges related to nonspecific drug distribution and toxicity to normal tissues. In biomedical detection and imaging, DNA assemblies can be engineered as precise “molecular hooks” to recognize specific disease biomarkers, achieving accurate detection of target molecules within complex biological systems. By incorporating fluorescent labeling, the developed fluorescent sensors can sensitively detect target molecules and are widely applied in real-time imaging of living cells. Furthermore, highly sensitive ECL detectors enable precise measurement of miRNAs, which is crucial for early tumor diagnosis. Plasmonic nanostructure-enhanced technologies have been employed in SERS-mediated biosensing and fluorescence spectroscopy-based biomolecule detection, achieving femtomolar-level sensitivity for miRNA detection. Enzyme-free signal amplification techniques such as CHA and HCR have shown great promise in these detection applications. Beyond nucleic acid detection, the analysis of telomerase, cancer cell proteins, and small molecules also plays a vital role in early tumor screening. As standalone nanosystems, DNA assemblies can serve both as carriers for therapeutic drugs and as detectors for diverse biomolecules. Although numerous challenges remain in this process, we firmly believe that DNA assemblies will emerge as one of the key tools for tumor diagnosis and treatment in the future.

## 6. Future prospects

Despite the impressive progress in DNA nanotechnology, its further development still faces a number of challenges and limitations. (1) Efficiency of delivery. DNA assemblies suffer from inefficiency in delivery, mainly due to insufficient membrane penetration and difficulty in release due to entrapment in lysosomes after endocytosis. For example, SNAs are endocytosed by the cell into the nuclear endosome and partially digested, resulting in a lack of their availability. To improve the lysosomal escape efficiency, Tian *et al.*^49^ designed an aggregation-induced emission (AIE) photosensitizer, which generates reactive oxygen species in the presence of light, destroying the lysosomal structure and promoting the escape of DNA nanomaterials. In addition to the traditional endocytosis pathway, non-endocytosis-dependent strategies have also received attention. For example, Li *et al.*^121^ developed DSMS, a bifunctional “molecular sticker” containing disulfide bonds and guanidino groups, which achieves direct cytoplasmic delivery of biomolecules through membrane fusion and sulfhydryl group-mediated uptake, maintains the structural integrity of the DNA assembly, exhibits high biocompatibility, reduces the risk of lysosome capture, and promotes efficient cellular uptake. (2) Stabilization. Although stable DNA assemblies have been developed, there is a need to further improve their stability, enzymatic resistance, and number of recognition sites. For example, DNA origami is susceptible to denaturation and dissociation in complex tumor microenvironments, affecting its drug-delivery ability. The main problem of SNAs is nuclease degradation, and the dissociability of their outer nucleic acids in different environments also limits their industrialization. For DNA hydrogels, enhancing their mechanical stability is necessary. It has been shown that the stability of DNA nanostructures can be enhanced by designing different coatings (*e.g.*, lipids, proteins, or peptides),^122^ oligonucleotide modification sites,^123^ or using different buffers.^124^ (3) The challenges for production. The fabrication process of DNA nanomaterials is complex and influenced by factors such as raw material purity and reaction conditions, leading to batch-to-batch variations. Additionally, the high cost and low yield of long-sequence DNA chemical synthesis limit their practical production and application. Future efforts should focus on cost reduction and efficiency improvement to achieve large-scale, precise manufacturing. (4) Long-term toxicity. Although modified DNA nanomaterials exhibit diverse functionalities, their long-term potential toxicity requires further investigation, including whether their degradation products harm organisms or trigger inflammation, immune responses, and genotoxicity. More clinical data are needed to support their applications. (5) Clinical translation of DNA assemblies. The enhanced permeability and retention (EPR) effect is nearly absent in advanced cancer patients. Chan W.C.W *et al.* found that only 0.7% of systemically administered NPs reach tumor sites, highlighting the inefficiency of EPR-based drug delivery.^125,126^ Furthermore, tumor size, type, and the TME influence the EPR effect. Larger tumors develop heterogeneous vasculature, restricting NP accumulation primarily to the tumor periphery.^127^ Tumor growth, driven by the Warburg effect, creates hypoxic and acidic conditions while activating inflammatory signaling cascades, increasing solid stress and interstitial fluid pressure.^127^ These factors collectively diminish the EPR effect in advanced cancers, reducing drug delivery efficiency and complicating the clinical translation of nanomedicines.

In the future, the application of DNA-assembled nanostructures in tumor diseases will continue to advance and develop. With technological progress, researchers will be able to innovate more stable, efficient, and safe DNA assemblies that perform critical functions in tumor detection and drug therapy. For example, designing DNA nanostructures with diverse surface modifications or increasing the ion concentration can enhance their mechanical strain resistance.^122^ The use of phage-generated monomer precursor DNA or asymmetric PCR techniques may reduce the cost of DNA origami. Additionally, by aligning with practical needs, DNA nanostructures can be applied across various fields to create customized forms such as nanorobots and nanodevices. With the advancement of nanotechnology, we are confident that DNA-assembled nanostructures will see widespread applications.

## Conflicts of interest

There are no conflicts to declare.

## Data Availability

All data presented in this review are derived from previously published studies, which have been appropriately cited throughout the manuscript. The figures included in this work have been reproduced with permission from the respective journals and publishers. Any further information regarding data sources can be obtained by referring to the original publications cited in the manuscript. As this is a review article, no new datasets were generated or analyzed specifically for this study.
